# Simvastatin inhibits stem cell proliferation in human leiomyoma via TGF‐β3 and Wnt/β‐Catenin pathways

**DOI:** 10.1111/jcmm.17211

**Published:** 2022-02-04

**Authors:** Sadia Afrin, Mohamed Ali, Malak El Sabeh, Qiwei Yang, Ayman Al‐Hendy, Mostafa A. Borahay

**Affiliations:** ^1^ Department of Gynecology and Obstetrics Johns Hopkins University School of Medicine Baltimore Maryland USA; ^2^ Clinical Pharmacy Department Faculty of Pharmacy Ain Shams University Cairo Egypt; ^3^ Department of Gynecology and Obstetrics University of Chicago School of Medicine Chicago Illinois USA

**Keywords:** proliferation, simvastatin, stem cell, TGF‐β3/SMAD2 signalling, uterine leiomyoma, Wnt4/β‐catenin signalling

## Abstract

Uterine leiomyoma (UL) is the most common gynaecologic tumour, affecting an estimated 70 to 80% of women. Leiomyomas develop from the transformation of myometrial stem cells into leiomyoma stem (or tumour‐initiating) cells. These cells undergo self‐renewal and differentiation to mature cells, both are necessary for the maintenance of tumour stem cell niche and tumour growth, respectively. Wnt/β‐catenin and TGF‐β/SMAD pathways, both overactive in UL, promote stem cell self‐renewal, crosstalk between stem and mature cells, cellular proliferation, extracellular matrix (ECM) accumulation and drive overall UL growth. Recent evidence suggests that simvastatin, an antihyperlipidemic drug, may have anti‐leiomyoma properties. Herein, we investigated the effects of simvastatin on UL stem cells. We isolated leiomyoma stem cells by flow cytometry using DyeCycle Violet staining and Stro‐1/CD44 surface markers. We found that simvastatin inhibits proliferation and induces apoptosis in UL stem cells. In addition, it also suppressed the expression of the stemness markers Nanog, Oct4 and Sox2. Simvastatin significantly decreased the production of the key ECM proteins, collagen 1 and fibronectin. Finally, it inhibited genes and/or proteins expression of TGF‐β1, 2 and 3, SMAD2, SMAD4, Wnt4, β‐Catenin, LRP6, AXIN2 and Cyclin D1 in UL stem cells, all are key drivers of the TGF‐β3/SMAD2 and Wnt4/β‐Catenin pathways. Thus, we have identified a novel stem cell‐targeting anti‐leiomyoma simvastatin effect. Further studies are needed to replicate these findings *in vivo*.

## 1 INTRODUCTION

Uterine leiomyomas (ULs) are the most common gynaecologic tumours, affecting approximately 70%–80% of women. They can cause heavy bleeding, pain and infertility.^1^ ULs are thought to originate from the transformation of myometrial stem cells into leiomyoma stem cells which, in turn, undergo division for self‐renewal which is necessary for the preservation of the stem cell niche; and differentiation to mature leiomyoma cells which contributes to tumour growth.^2^

Leiomyoma stem cells, also called tumour‐initiating cells, express high levels of stemness markers including Nanog, Oct4, Sox2 and KLF4, while showing low levels of oestrogen receptor, progesterone receptor and smooth muscle actin, in line with their undifferentiated status.^3^, ^4^ UL stem cells also have a shorter doubling time than myometrial progenitor cells.^3^ Several studies have proposed that a single myometrial stem cell undergoes tumorigenic transformation following hypoxia, *MED12* mutations, abnormal methylation and/or aberrant steroid signalling.^5^, ^6^, ^7^

Leiomyoma stem cells have been identified using flow cytometry.^8^ Stem cells highly express the transporter ABCG2 which rapidly pumps out cell‐permeable DNA‐binding dyes such as Hoechst 33342 and DyeCycle Violet (DCV).^9^ Therefore, these cells appear dim and are identified as the side population (SP) on flow cytometry. Cells that do not exclude (efflux) the dye are called main population (MP) and are mostly mature non‐stem cells. Thus, certain fluorescent DNA stains can separate total leiomyoma cell population cells into SP that includes stem cells and MP that includes mature cells. In addition, specific cell surface markers such as Stro‐1 and CD44 have been used to identify myometrial and leiomyoma stem cells since cells displaying these markers possess stem or progenitor cell features.^4^

Tumorigenic transformation of myometrial cells is known to be associated with overactive Wnt/β‐catenin pathway.^10^, ^11^ The Wnt/β‐catenin pathway has been shown to be essential for the interaction between leiomyoma stem cells and the neighbouring mature cells and tumour responsiveness to oestrogen and progesterone.^12^, ^13^, ^14^ Additionally, this pathway stimulates transforming growth factor‐beta (TGF‐β) expression, which promotes cell proliferation and extracellular matrix (ECM) formation.^15^, ^16^ Mutations of *MED12* increase TGF‐β receptor expression, which leads to aberrant activation of SMAD and MAPK, altering stem cell self‐renewal, proliferation and fibrosis.^17^

Even though ULs are the most common cause of hysterectomies, current treatment options for UL are limited.^18^ An optimal treatment strategy would target the pathways implicated in the development and proliferation of leiomyoma stem cells, the origin of UL. Simvastatin, an antihyperlipidemic drug, has been widely used for treating hypercholesterolemia, atherosclerosis and coronary artery disease for more than 20 years.^19^ Evidence suggests it may have a therapeutic role in leiomyoma. In a nested case–control study, statin users had a lower risk and symptoms of leiomyoma than nonusers.^20^ In uterine leiomyoma cells, simvastatin exerts beneficial effects through inhibiting cell proliferation, arresting cell cycle,^21^, ^22^ inducing apoptosis and reducing ECM production.^23^ In addition, simvastatin significantly decreases leiomyoma growth in a patient‐derived xenograft mouse model.^21^ Based on the above studies, simvastatin might be a promising candidate for the treatment of uterine leiomyoma.^24^

In the present study, we sought to examine the effects of simvastatin on leiomyoma tumour‐initiating cells. We found that simvastatin inhibits proliferation, induces apoptosis, reduces the expression of key pathways involved in promoting stem cell proliferation, stemness, fibrosis, ECM accumulation and intracellular signalling. Thus, we identified a novel stem cell‐targeting anti‐leiomyoma therapeutic mechanism.

## 2 MATERIALS AND METHODS

### 2.1 Human specimen collection and sample preparation

Leiomyoma tissues were collected from patients who underwent hysterectomy at the Department of Gynecology and Obstetrics at the Johns Hopkins Hospital, Baltimore, MD, USA and University of Illinois at Chicago, IL, USA. The Johns Hopkins University and University of Illinois Institutional Review Boards reviewed and approved the study and informed consents were obtained from patients. Buffer containing Hanks' Balanced Salt Solution (HBSS, Thermo Fisher Scientific, Waltham, MA) and 1% antibiotic–antimycotic solution (Thermo Fisher Scientific) were used to wash tissues. Tissues were manually cut into small pieces (<1 mm^3^) and incubated in sterile HBSS (without phenol, calcium or magnesium) with collagenase (Worthington), deoxyribonuclease (DNase, Sigma‐Aldrich), antibiotic‐antimycotic mixture and HEPES buffer solution (Thermo Fisher Scientific) on a shaker for 4 to 8 h at 37°C to digest the tissue.

### 2.2 Chemicals

Simvastatin, Pravastatin, Lovastatin, Atorvastatin and Fluvastatin (Cayman Chemical, Ann Arbor, MI, USA) stock solutions (10 mM) were prepared in dimethyl sulfoxide (DMSO; Sigma‐Aldrich) and stored at −20°C until use. The rest of the chemicals and reagents used in the experiments were purchased from Sigma‐Aldrich and Thermo Fisher Scientific.

### 2.3 Isolation of stem cells side population and antibody‐based sorting by FACS

Flow cytometry using DCV and Stro‐1 and CD44 antibodies was used to isolate stem cells from fresh human leiomyomas from 5 patients as previously described^8^ with minor modifications. Briefly, the tissue digest was filtered through a 100 µm filter and resuspended at a concentration of 1 × 10^6^ cells/ml in calcium‐ and magnesium‐free Hanks‐balanced salt solution (HBSS) containing 2% foetal bovine serum (FBS). DCV (Invitrogen, Waltham, MA) was then added at a final concentration of 5 µM, and the sample was incubated at 37° C for 90 min with gentle vortexing every 15 min to allow maximum dye penetration. Extracellular DCV was washed away in 5x volume ice‐cold PBS, resuspended in 1 ml of cold FACS solution, and further incubated with 2 μg/ml propidium iodide (PI, Sigma‐Aldrich) to label the nonviable cells. The cells were always kept on ice after staining and were subjected to flow cytometric analysis by FACS‐Aria (BD Bioscience) to separate the SP and MP. After DCV staining, cells were immunolabelled with antibodies prior to flow cytometry. The cells were centrifuged for 7 min at 400 × g, 4°C and resuspended in HBSS. The desired biotinylated and conjugated antibodies to Stro‐1 (R&D Systems, Minneapolis, MN, USA) and CD44 (BD Biosciences) were added for 30 min on ice in the dark. The excess antibody was removed after washing with HBSS. Cells were sorted by BD FACSCalibur flow cytometer system (Beckman‐Coulter), and data were analysed using FlowJo software (Tree Star Inc.). In this work, stem cells were identified as SP cells on DCV staining that were also Stro‐1^+^/CD44^+^. For our experiments, we used stem cells isolated from different patients.

### 2.4 Cell culture and treatment

In our previous publications, the proliferation rate of leiomyoma stem cells was measured to determine the effect of FBS concentration.^25^ At 24, 48 and 72 h, with DMEM/F12 medium was supplemented with 0%, 3%, 6% or 12% FBS, leiomyoma stem cells did not proliferate differently from myometrium stem cells. Since we found that our cells grew well in DMEM/F12 medium containing 12% FBS and were able to complete previous experiments under this condition, we used this level of FBS supplementation in all in vitro experiments. We cultured leiomyoma stem cells (Stro‐1^+^/CD44^+^) in DMEM/F12 (Thermo Fisher Scientific) medium supplemented with 12% FBS, and 1% antibiotic‐antimycotic in collagen‐coated dishes in a humidified atmosphere with 2% O2 and 5% CO_2_ at 37°C. The mature cells (Stro‐1^−^/CD44^−^) and primary leiomyoma (total cell population including both stem and mature) cells were separately cultured in DMEM/F12 medium with 10% FBS, and 1% antibiotic‐antimycotic in a humidified atmosphere with regular 2% O2 and 5% CO_2_ at 37°C. Cells were maintained and treated with simvastatin or DMSO (vehicle control) for 48 h.

### 2.5 Quantitative RT‐PCR

RNA was extracted using the RNeasy plus mini kit (QIAGEN, Gaithersburg, MD) following the manufactures' protocol. cDNA was synthesized in a Bio‐Rad Thermocycler using the iScript cDNA Synthesis Kit (Bio‐Rad) according to the manufacturer's instructions. LightCycler 96 System (Roche Diagnostics) and FastStart Essential DNA Green Master (Roche Diagnostics) were used for the qRT‐PCR. The relative mRNA expression was expressed as fold change and calculated using the 2^∆∆CT^ method by using specific primers (Table S1).

### 2.6 Immunoblotting

Leiomyoma stem cells were treated with simvastatin (0.01, 0.1, 1 µM) or DMSO (vehicle control) for 48 h. Cells were lysed in a lysis buffer (radioimmunoprecipitation assay buffer, Sigma‐Aldrich) containing a protease and phosphatase inhibitor cocktail (Sigma‐Aldrich). An equal amount of protein lysates was loaded on 4 to 12% Bis‐Tris protein gradient gels (Thermo Fisher Scientific) and transferred to a nitrocellulose membrane (Thermo Fisher Scientific) using the mini trans‐blot transfer system (Thermo Fisher Scientific). To detect specific antigens, blots were probed with primary antibodies (Table S2) on a shaker at 4° C overnight, followed by 1 h of room temperature incubation with HRP conjugated secondary antibodies (GE Healthcare). Chemiluminescent Western blot signal was captured was using an Azure Imager c300 (Azure Biosystems). Band signals were quantified with the NIH ImageJ software (version 1.52r).

### 2.7 Immunocytochemistry

Cells were cultured and treated on the glass slides, then fixed with 4% formaldehyde and incubated in a blocking solution (1× PBS, 5% normal goat serum (CST), and 0.3% Triton X‐100 (Sigma‐Aldrich) for 1 h at room temperature. Primary antibodies (Table S2) were incubated overnight at 4°C. Next, we used Alex488‐conjugated anti‐rabbit (Invitrogen, #A11034) and Alex594‐conjugated anti‐mouse (Invitrogen, #A11030) secondary antibodies for further staining. A ProLong Gold Antifade reagent with DAPI (Thermo Fisher Scientific) was used to fix the slide overnight at room temperature. The samples were examined using a Leica SP8 Microsystems (Wetzlar, Germany) confocal microscope. As each image had 4 megapixels and the time of acquaintance was 1.9 microseconds per pixel, the total scanning time was 7.6 s.

### 2.8 Viability assays

Leiomyoma stem cells were plated onto a 96‐well plate and allowed to grow overnight. Cells were treated with different statins at the indicated concentrations or DMSO (vehicle control) for 48 h. Concentrations were chosen based on previous publications and clinical relevance.^21^, ^22^, ^26^ Viability was assessed by (3‐[4,5‐dimethylthiazol‐2‐yl]‐2,5‐diphenyltetrazolium bromide) (MTT) staining by measuring absorbance at 500–600 nm.

### 2.9 Caspase‐3 assay

Cells were treated with different simvastatin doses for 48 h and DMSO as vehicle control. Caspase‐3 activity was detected using a quantitative fluorometric assay as previously described.^27^

### 2.10 Hoechst 33342 Staining for apoptosis

Leiomyoma stem cells were seeded onto coverslips in a 6‐well plate and treated with simvastatin (1 μM). After 48 h, the attached cells were washed with PBS and fixed in freshly prepared 4% paraformaldehyde for 30 min, then washed with PBS and incubated with Hoechst 33342 staining solution for 5 min. Cells were then washed with PBS, an antifade mounting medium, then apoptosis was detected using fluorescence microscope.

### 2.11 Statistical analysis

Data were analysed using GraphPad Prism version 6.01 for Windows (GraphPad). D’Agostino–Pearson omnibus normality test was used for data distribution. Unpaired Student's t‐test and one‐way ANOVA with an appropriate Tukey's post hoc test were used according to the type of experiment. The nonparametric Mann–Whitney U and Kruskal–Wallis followed by Dunn's test were used for mRNA and protein expression levels, respectively. All experiments were performed in duplicate and repeated three independent times from different patients. Error bars represent a standard error of the mean (SEM). *p*‐values <0.05 were considered significant (*p < 0.05; **p < 0.01; ***p < 0.001); *p* > 0.05, non‐significant (NS).

## 3 RESULTS

### 3.1 Isolation and characterization of the human leiomyoma Stro‐1^+^/CD44^+^ stem cells

**FIGURE 1:**
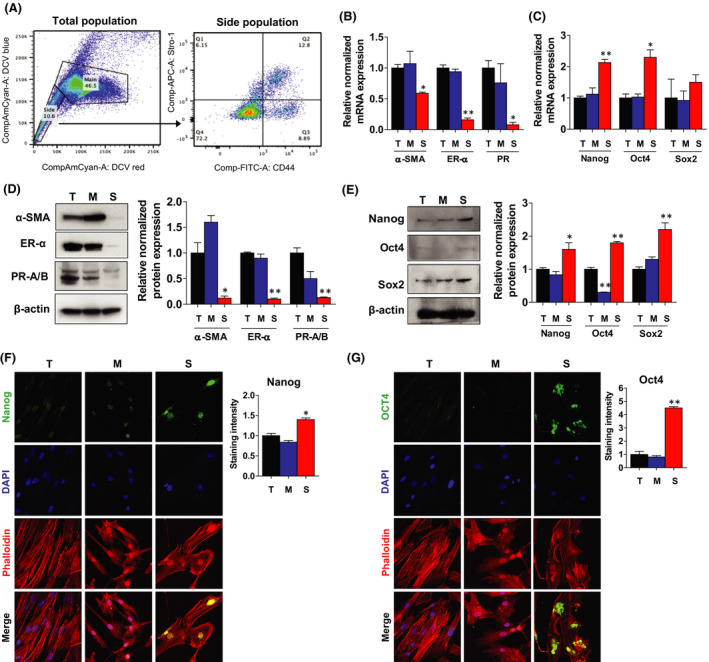
Isolation and characterization of the human leiomyoma (Stro‐1+/CD44+) stem cells. A Left, distribution of the side population (SP) and main population (MP) cells within DCV‐stained living cells isolated from human leiomyoma tissue. The representative experiment was repeated using cells from five independent subjects. Right, representative flow cytometric profile of SP cells based on the expression pattern of Stro‐1 and CD44. B, C The mRNA expression levels of smooth muscle cell marker (*α*‐*SMA*), steroid receptors (*ERα and PR*) and stemness transcription factors (*Nanog*, *Oct4 and Sox2*) were quantitated by RT‐PCR in the total leiomyoma cell population (T) and the two FACS‐sorted subpopulations: mature (M) and stem (S) cells. *RPLP0* was amplified under the same RT‐PCR conditions and used for normalizing data. D, E α‐SMA, ESR1, PR‐A/B, Nanog, Oct4 and Sox2 protein expression levels were detected by Western blotting, and β‐actin was used as a loading control between three leiomyoma cell populations (T, M and S). F, G Immunofluorescence staining of T, M and S cells isolated from leiomyoma tissues with antibodies against Nanog (green) and Oct4 (green). Nuclei were stained with DAPI (blue), and actin was stained with phalloidin (red). All images were captured with the same time exposure using a confocal microscope (20 × magnification). Data are presented as the means ±standard error of the mean (SEM) of the relative expression obtained from three independent experiments. *p < 0.05; **p < 0.01

Fresh leiomyoma tissue was collected from patients, and a single‐cell suspension was prepared. FACS was performed using DCV stain to separate total cells into SP (10.6%) and MP (mature leiomyoma cells) (46.6%) fractions. The SP fraction was further separated using Stro‐1 and CD44 antibodies. The percentages of the SP subpopulations Stro‐1^+^/CD44^+^ and Stro‐1^−^/CD44^−^ were 12.8% and 72.2%, and intermediate cell populations, Stro‐1^+^/CD44^−^ and Stro‐1^−^/CD44^+^ were 6.15% and 8.89% of the, respectively, (Figure 1A **)**. In this work, stem cells were defined as Stro‐1^+^/CD44^+^.

To determine the expression of stemness markers and steroid hormone receptors of the isolated stem cells (Stro‐1^+^/CD44^+^), we performed RT‐PCR, Western blot and immunocytochemistry (ICC) analysis. The mRNA isolated from total, mature and stem cells confirmed the expression levels of *α*‐*SMA*, *ESR1* and *PR*, and the expression levels were significantly greater in total and mature cells than in stem cells **(**Figure 1B **)**. The results also confirmed at the protein level as well (Figure 1D). There are no significant differences between the mature and total cells in their expression of α‐SMA, ESR1 and PR. The mRNA and protein expression of typical undifferentiated stem cell‐related markers, *Nanog*, *Oct4* and *Sox2*, were highly expressed in stem cells compared to total and mature cells (Figure 1C, E**)**. However, there was no difference in mRNA *Sox2* levels. The ICC analysis further revealed that stem cells exhibited greater immunostaining intensity for Nanog and Oct4 than total and mature cells (Figure 1F,G).

Our previous publication reported that leiomyoma stem cells grow optimally in DMEM/F12 media with 12% FBS.^9^ ABCG2 transporter expression, Oct4, Nanog and GDB3 expression, along with the low expression of ER‐α and PR‐A/PR‐B receptors, confirmed the undifferentiated status of isolated cells. These cells did not contain CD34 or CD45, indicating their mesodermal origin, and they were able to differentiate into adipocytes, osteocytes and chondrocytes in vitro. Lastly, they made fibroid‐like lesions in a mouse xenotransplantation model.

In the present study, the expression of typical undifferentiated stem cell‐related genes was validated by qRT‐PCR, Western blot and ICC. As shown in Figure 1, Stro‐1+/CD44+ human stem cells have a distinct molecular profile versus mature and total cell population.

### 3.2 Antiproliferative and pro‐apoptotic effects of simvastatin on leiomyoma stem cells

**FIGURE 2:**
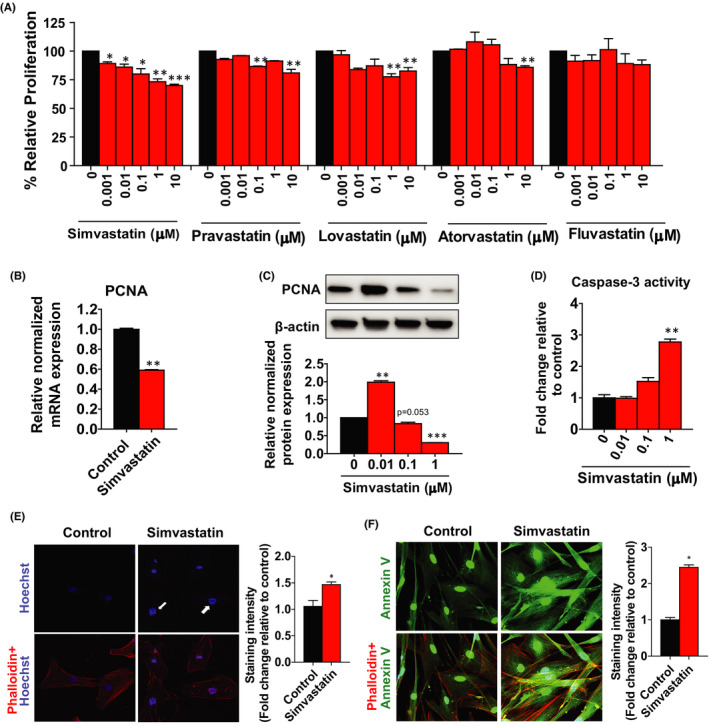
Antiproliferative and apoptotic effects of simvastatin. A Human uterine leiomyoma stem cells were treated with simvastatin, pravastatin, lovastatin, atorvastatin and fluvastatin at different doses (0.001, 0.01, 0.1, 1 and 10 µM) for 48 h. Dimethyl sulfoxide (DMSO) was used as vehicle control. Cell proliferation was measured by an MTT assay. B The mRNA expression levels of *PCNA* were quantitated by RT‐PCR after simvastatin (1 µM) treatment for 48 h. *RPLP0* was amplified under the same RT‐PCR conditions used for normalizing data. C PCNA protein expression levels were detected by Western blotting after simvastatin (0.01, 0.1, 1 µM) treatment for 48 h. β‐actin was used as a loading control. D Caspase‐3 enzymatic activity showed that simvastatin (0.01, 0.1, 1 µM) treatment for 48 h increases apoptotic signalling in leiomyoma stem cells. Nuclear staining with Hoechst 33342 (blue) (E) and Annexin V (green) (F) showed that simvastatin treatment for 48 h increases fragmented and condensed apoptotic nuclei compared to control. Actin was stained with phalloidin (red). All images were captured with the same time exposure using a confocal microscope (20× magnification). The same Western blot membrane was probed for PCNA, Collagen 1 and β‐catenin, presented in Figures 2C, 4B and 7E, respectively. Therefore, the same β‐actin image was used for all of them. Data are presented as the means ±SEM of the relative expression obtained from three independent experiments. *p < 0.05; **p < 0.01; ***p < 0.001

To explore whether statins have any effect on leiomyoma stem cell proliferation, we examined the antiproliferative effects of five different types of statins, including simvastatin, pravastatin, lovastatin, atorvastatin and fluvastatin at different concentrations (0.001, 0.01, 0.1, 1 and 10 µM) for 48 h. Results demonstrated that simvastatin at all doses significantly decreases the viability of leiomyoma stem cells in a dose‐dependent manner with a growth inhibitory effect of 21 to 30% (Figure 2A). Simultaneously, pravastatin, lovastatin and atorvastatin reduced cell viability by 7 to 19%, 3 to 17% and 12 to 14%, respectively, while fluvastatin had no significant effect at the concentration tested (Figure 2A). We used simvastatin for further experiments based on the data mentioned above. After simvastatin treatment for 48 h, the mRNA (Figure 2B) and protein (Figure 2C) levels of proliferation‐related marker PCNA were significantly decreased by 1.67‐fold and 2.22‐fold, respectively, compared to untreated cells.

In response to simvastatin treatment, we examined the caspase‐3 enzyme activity to determine if it could induce apoptosis in addition to the inhibition of cell growth. Simvastatin treatment of 1µM significantly upregulated caspase‐3 activity by 3.00‐fold in leiomyoma stem cells compared to untreated control **(**Figure 2D). This finding was confirmed by simvastatin treatment which increased nuclear Hoechst 33342 staining by 1.5‐fold and annexin V apoptosis staining by 2.25‐fold, an indicator of fragmented and condensed apoptotic nuclei (Figure 2E,F).

### 3.3 Simvastatin effect on stemness markers

**FIGURE 3:**
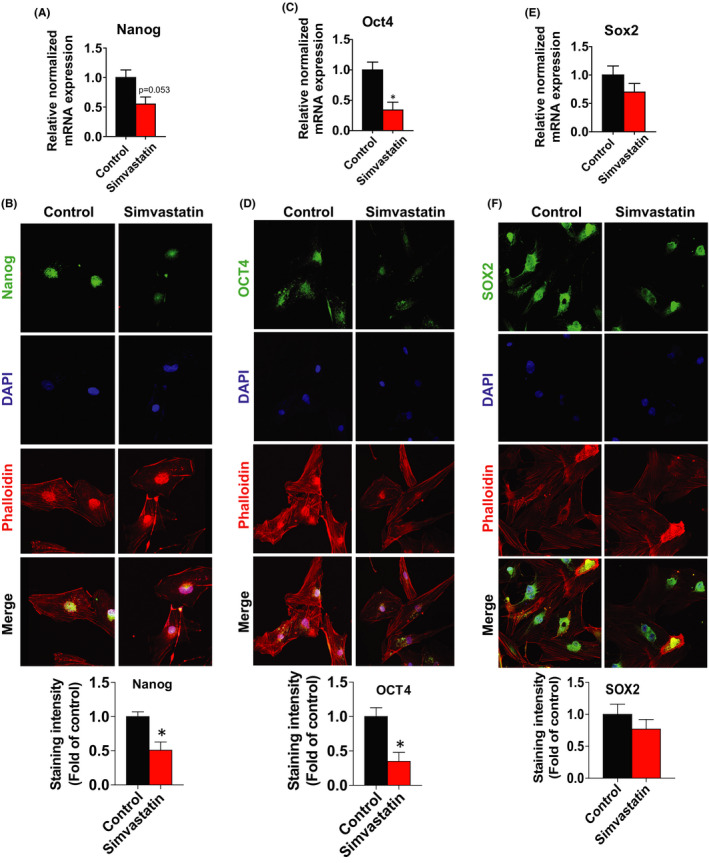
Effect of simvastatin on stemness transcription factors. A, C, E Human uterine leiomyoma stem cells were treated with simvastatin (1 µM) for 48 h. DMSO was used as vehicle control. The mRNA expression levels of *Nanog*, *Oct4* and *Sox2* were quantitated by RT‐PCR. *RPLP0* was amplified under the same RT‐PCR conditions used for normalizing data. B, D, F Immunofluorescence staining was performed on leiomyoma stem cells with an antibody against Nanog (green), Oct4 (green) and Sox2 (green) after simvastatin (1 µM) treatment for 48 h. Nuclei were stained with DAPI (blue), and actin was stained with phalloidin (red). All images were captured with the same time exposure using a confocal microscope (20 × magnification). Data are presented as the means ±SEM of the relative expression obtained from three independent experiments. *p < 0.05; **p < 0.01; ***p < 0.001

Studies have shown that transcription factors such as Nanog, Oct4 and Sox2 play a vital role in the pluripotency, self‐renewal regulation, differentiation status and maintenance of stem cell phenotypes.^28^ The reprogramming of somatic stem cells into pluripotent stem cells by upregulation of these factors drastically increases tumorigenesis ability.^29^ To explore whether simvastatin can affect Nanog, Oct4 and Sox2 expressions, we performed RT‐PCR and ICC analysis. Simvastatin treatment reduced the mRNA levels of *Oct4* (2.94‐fold), *Nanog* (1.81‐fold) and *Sox2* (1.43‐fold) compared to control (1.00‐fold) **(**Figure 3A,C,E**)**. ICC analysis revealed that all tested transcription factors were reduced in the nuclei of leiomyoma stem cells following simvastatin treatment (Figure 3B,D,F).

### 3.4 Suppression of fibrosis‐related gene expression after simvastatin treatment

**FIGURE 4:**
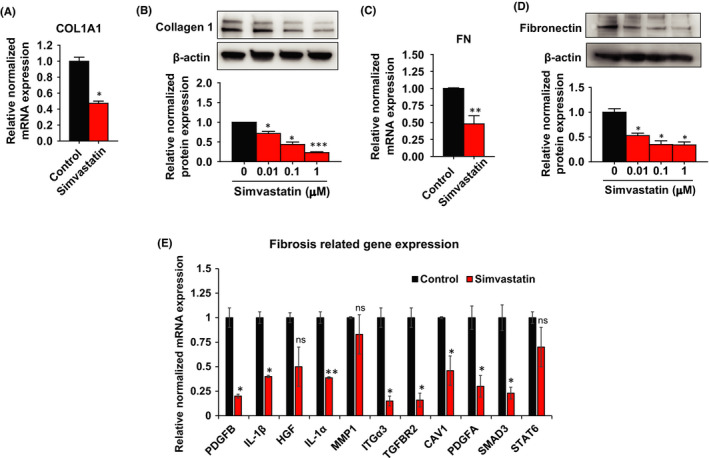
Suppression of fibrosis‐related gene expression after simvastatin treatment. Human uterine leiomyoma stem cells were treated with simvastatin (1 µM) for 48 h. DMSO was used as vehicle control. A, C The mRNA expression levels of collagen 1 (*COL1A1*), and fibronectin (*FN*) were quantitated by RT‐PCR. *RPLP0* was amplified under the same RT‐PCR conditions and used for normalizing data. B, D Collagen 1 and fibronectin, protein expression levels were detected by Western blotting after simvastatin treatment (0.01, 0.1, 1 µM) for 48 h. β‐actin was used as a loading control. E The mRNA levels of fibrosis‐related genes were quantitated by RT‐PCR. *RPLP0* was amplified under the same RT‐PCR conditions and used for normalizing data. The same Western blot membrane was probed for PCNA, Collagen 1 and β‐catenin, presented in Figures 2C, 4B and 7E, respectively. Therefore, the same β‐actin image was used for all of them. Data are presented as the means ±SEM of the relative expression obtained from three independent experiments. *p < 0.05; **p < 0.01; ***p < 0.001

Fibrosis plays a crucial mediator in UL growth. This condition is commonly characterized by ECM accumulation and excessive production of inflammatory cytokines and chemokines.^30^, ^31^ To see whether simvastatin altered the expression of fibrosis‐related genes, we checked the expression of collagen 1 and fibronectin, which are essential ECM proteins overexpressed in the ULs. Simvastatin treatment significantly suppressed the expression of both mRNAs (2.13‐fold) and protein (1.41‐fold to 4.34‐fold) levels of collagen 1 in stem cells (Figure 4A,B). Similarly, we observed a significant reduction of both mRNA and protein expression levels of fibronectin after simvastatin treatment (Figure 4C,D).

To explore whether simvastatin can affect the expression of more fibrosis‐related genes, we performed RT‐PCR assay after 48 h of treatment and compared it to untreated stem cells (Figure 4E). The results indicated that simvastatin remarkably decreases the expression of a large number of genes that are implicated in fibrosis development, including platelet‐derived growth factor subunit (*PDGFB*, *PDGFA*), inflammatory cytokines (*IL*‐*1β*, *IL*‐*1α*), matrix metalloproteinase (*MMP1*), membrane proteins (*CAV1*, *ITGα3*), growth factors (HGF, TGF‐β subunits), growth factor signalling (*SMAD3*) and transcription factors (*STAT6*) while there was no change in *AKT1*, *MMP14*, *MMP3*, *CTGF*, *CCL3*, *SMAD6* and *STAT1* (data not shown).

### 3.5 Simvastatin reduced TGF‐β3/SMAD2 signalling in leiomyoma stem cells

TGF‐β is widely recognized as a potent inducer of fibrosis considering its involvement in various ECM expression and deposition^16^, ^32^ and has been shown to be overexpressed in ULs.^33^

**FIGURE 5:**
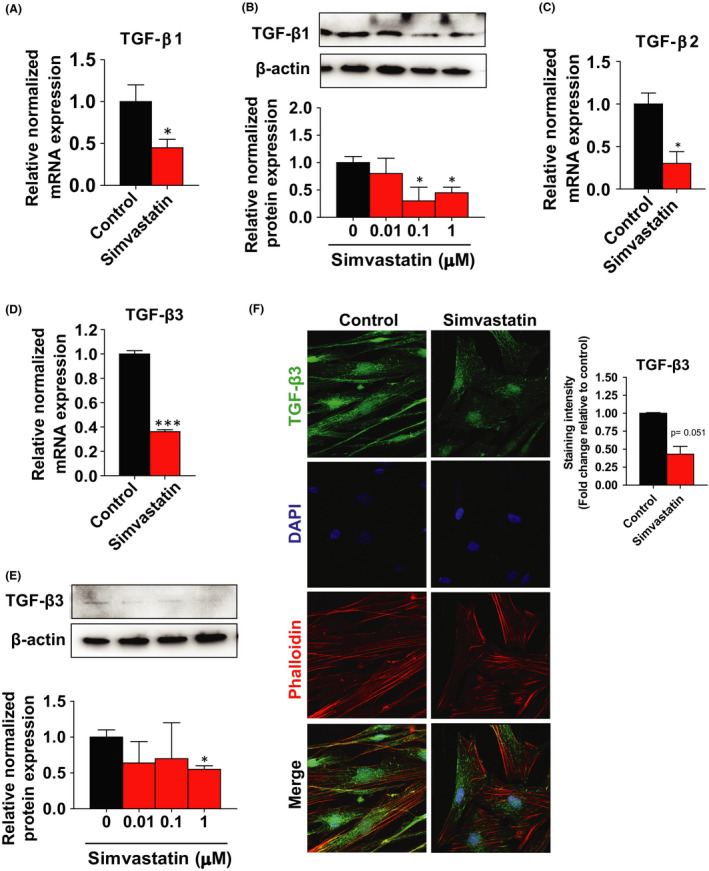
Inhibition of TGF‐β signalling after simvastatin treatment. A, C, D Human uterine leiomyoma stem cells were treated with simvastatin (1 µM) for 48 h. DMSO was used as vehicle control. The mRNA expression levels of *TGF*‐*β1*, *TGF*‐*β2* and *TGF*‐*β3* were quantitated by RT‐PCR. *RPLP0* was amplified under the same RT‐PCR conditions and used for normalizing data. B, E TGF‐β1 and TGF‐β3 protein expression levels were detected by Western blotting after simvastatin (0.01, 0.1, 1 µM) treated for 48 h. β‐actin was used as a loading control. F Immunofluorescence staining was performed on leiomyoma stem cells with an antibody against TGF‐β3 (green) after simvastatin (1 µM) treatment for 48 h. Nuclei were stained with DAPI (blue), and actin was stained with phalloidin (red). All images were captured with the same time exposure using a confocal microscope (20× magnification). Data are presented as the means ±SEM of the relative expression obtained from three independent experiments. *p < 0.05; **p < 0.01; ***p < 0.001

In this study, we found that simvastatin treatment remarkably reduces the transcription levels of *TGF*‐*β1*, *TGF*‐*β2* and *TGF*‐*β3* by 2.04‐fold, 3.33‐fold and 2.78‐fold in leiomyoma stem cells compared to untreated control (Figure 5A,C,D). In agreement with the transcript results, simvastatin treatment showed a reduction in TGF‐β1 (2.00 – 2.86‐fold) and TGF‐β3 (1.67‐fold) protein expression after 48 h of treatment (Figure 5B,E). Next, we observed the effects of simvastatin on the staining intensity of TGF‐β3, where simvastatin treatment also suppressed the TGF‐β3 expression level **(**Figure 5F). Additionally, the protein expression levels of TGF‐β1, 2 and 3 in mature and stem cells confirm the significance of these cytokines in the fibrotic process. As shown in Figure S1, there are no differences in the expression levels of TGF‐β1, 2 and 3 between the mature and stem cells.

**FIGURE 6:**
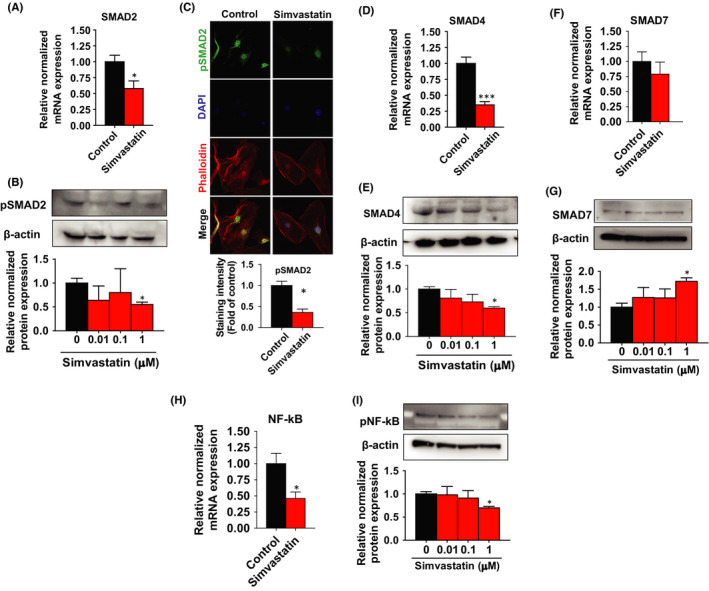
Inhibition of SMAD signalling after simvastatin treatment. A, D, F, H Human uterine leiomyoma stem cells were treated with simvastatin (1 µM) for 48 h. DMSO was used as vehicle control. The mRNA expression levels of *SMAD2*, *SMAD4*, *SMAD7* and *NF*‐*κB* were quantitated by RT‐PCR. *RPLP0* was amplified under the same RT‐PCR conditions and used for normalizing data. B, E, G, I pSMAD2/SMAD2, SMAD4, SMAD7 and pNF‐κBp65/NF‐κBp65 protein expression levels were detected by Western blotting after simvastatin (0.01, 0.1, 1 µM) treated for 48 h. β‐actin was used as a loading control. C Immunofluorescence staining was performed on leiomyoma stem cells with an antibody against pSMAD2 (green) after simvastatin (1 µM) treatment for 48 h. Nuclei were stained with DAPI (blue), and actin was stained with phalloidin (red). All images were captured with the same time exposure using a confocal microscope (20 × magnification). Data are presented as the means ±SEM of the relative expression obtained from three independent experiments. *p < 0.05; **p < 0.01; ***p < 0.001

TGF‐β binds to its transmembrane receptor on the cell surface and activates downstream SMAD2 and SMAD3, which in turn binds with SMAD4. SMAD4 is then translocated into nucleus where it induces of pro‐fibrotic genes.^34^ In this study, simvastatin significantly suppressed *SMAD2* mRNA levels (1.72‐fold) in leiomyoma stem cells (Figure 6A). Moreover, since phosphorylation of SMAD2 is required for its activation, simvastatin treatment significantly decreased the protein expression levels by 1.67‐fold (Figure 6B) and staining signals by 2.5‐fold (Figure 6C) of phosphorylated SMAD2. We also detected a 2.85‐fold and 1.80‐fold reduction of SMAD4 mRNA and protein levels by simvastatin treatment (Figure 6D,E). The inhibitory SMAD7 interacts with TGF‐β receptors and obstructs phosphorylation of SMADs, disturbing TGF‐β signalling.^35^ Our experiment detected a non‐significant decreased mRNA and a significantly increased protein expression of SMAD7 in response to simvastatin treatment in leiomyoma stem cells (Figure 6F,G). Prior studies have shown that SMAD signalling induces inflammation by activating NF‐κB dependent inflammatory responses. Simvastatin significantly decreased the mRNA levels of NF‐κB by 2.00‐fold and the protein levels of phosphorylation of NF‐κBp65 by 1.54‐fold in leiomyoma stem cells compared to control (Figure 6H,I), suggesting the reduction of SMAD7 activated inflammation may be via NF‐κB dependent pathway.^36^

### 3.6 Inhibition of Wnt4/β‐catenin pathway by simvastatin

When the Wnt ligand is absent, β‐catenin undergoes phosphorylation by the destruction complex, consisting of Axin, APC, GSK3‐β and CK1. This leads to β‐catenin ubiquitination and subsequent proteasomal degradation. However, in the presence of the Wnt ligand, β‐catenin is released from the destruction complex, accumulates in the cytosol. The accumulated β‐catenin is then translocated into the nucleus. β‐catenin interacts with LEF/TCF transcription factors and regulates the expression of an enormous number of genes, including cyclin D1. A comparison of the Figure S2 indicates slightly higher levels of Wnt4 expression in stem cells compared with the mature cells. Earlier reports showed Wnt4 was primarily expressed in leiomyoma intermediate and differentiated cells, while FZD6 was predominantly expressed in stem cells.^37^

**FIGURE 7:**
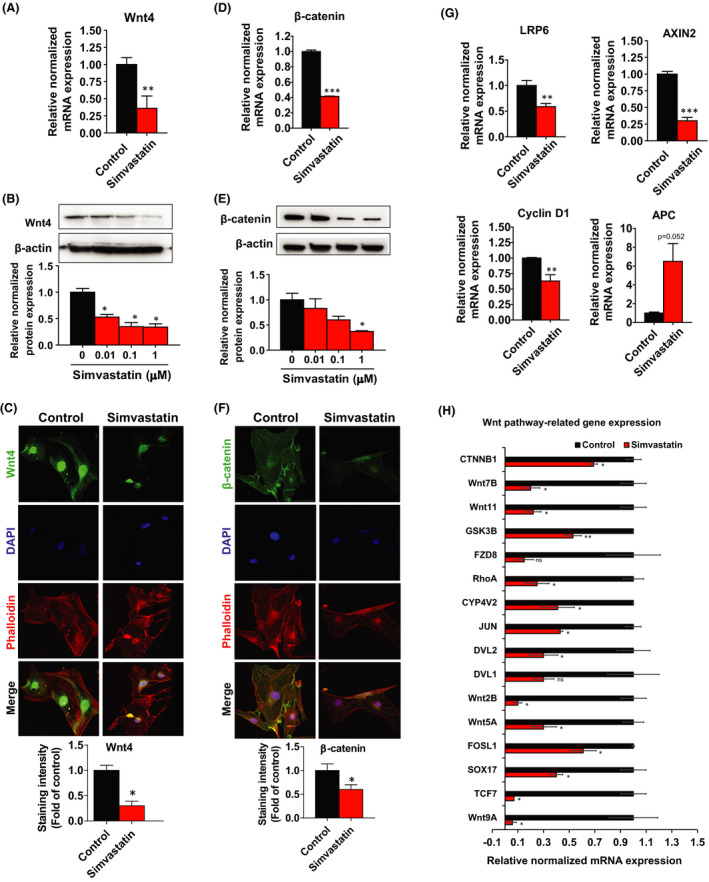
Inhibition of Wnt/β‐Catenin pathway after simvastatin treatment. A, D, G Human uterine leiomyoma stem cells were treated with simvastatin (1 µM) for 48 h. DMSO was used as vehicle control. The mRNA expression levels of *Wnt4*, *β*‐*catenin LRP6*, *AXIN2*, *Cyclin D1* and *APC* were quantitated by RT‐PCR. *RPLP0* was amplified under the same RT‐PCR conditions used for normalizing data. B, E Wnt4 and β‐catenin protein expression levels were detected by Western blotting after simvastatin (0.01, 0.1, 1 µM) treated for 48 h. β‐actin was used as a loading control. C, F Immunofluorescence staining was performed on leiomyoma stem cells with an antibody against Wnt4 (green) and β‐catenin (green) after simvastatin (1 µM) treatment for 48 h. Nuclei were stained with DAPI (blue), and actin was stained with phalloidin (red). All images were captured with the same time exposure using a confocal microscope (20 × magnification). H, The Wnt signalling‐associated gene's levels were detected using RT‐PCR assay after simvastatin (1 µM) treatment for 48 h. *RPLP0* was amplified under the same RT‐PCR conditions and used for normalizing data. The same Western blot membrane was probed for PCNA, Collagen 1 and β‐catenin, presented in Figures 2C, 4B and 7E, respectively. Therefore, the same β‐actin image was used for all of them. Data are presented as the means ±SEM of the relative expression obtained from three independent experiments. *p < 0.05; **p < 0.01; ***p < 0.001

To examine whether simvastatin affects the expression of genes related to β‐catenin and Wnt4, which has been previously shown to be overexpressed in UL.^38^ In leiomyoma stem cells, we performed RT‐PCR, Western blot and ICC analysis. Simvastatin treatment robustly decreased mRNA levels of *Wnt4* (2.78‐fold) **(**Figure 7A **)** and *β*‐*catenin* (2.38‐fold) **(**Figure 7D **)** compared to control. In agreement with the mRNA data, simvastatin significantly suppressed the protein levels of Wnt4 and β‐catenin in a dose‐dependent manner (Figure 7B,E). Wnt4 immunostaining intensity was mainly located in the nuclei, and very little was present in the cytoplasm of leiomyoma stem cells, where simvastatin treatment weekend nuclei signals **(**Figure 7C). ICC analysis showed β‐catenin expression in both the cytoplasm and the nuclei of leiomyoma stem cells; simvastatin efficiently decreased β‐catenin staining signals in both compartments of the cells **(**Figure 7F).

We also measured the mRNA levels of *LRP6*, *AXIN2*, *Cyclin D1* and *APC* by RT‐PCR. Simvastatin treatment significantly inhibited transcript levels of *LRP6* (1.69‐fold), *AXIN2* (3.33‐fold) and *Cyclin D1* (1.59‐fold) **(**Figure 7G), while we found a higher expression of the tumour suppressor APC levels compared to control.

Furthermore, we performed screening to evaluate the simvastatin effect on the other Wnt signalling‐associated genes on leiomyoma stem cells by using the RT‐PCR. Treatment with simvastatin remarkably decreased the expression levels of a large panel of genes including *CTNNB1*, *Wnt9A*, *Wnt5B*, *Wnt2B*, *Wnt7B*, *Wnt11*, *TCF7*, *SOX17*, *FOSL1*, *DVL2*, *JUN*, *RhoA* and *GSK3β* compared to untreated control **(**Figure 7H) while *Wnt2*, *Wnt6*, *Wnt10A* and *FZD4*, *5*, *AXIN1* genes were unchanged (data not shown).

## 4 DISCUSSION

**FIGURE 8:**
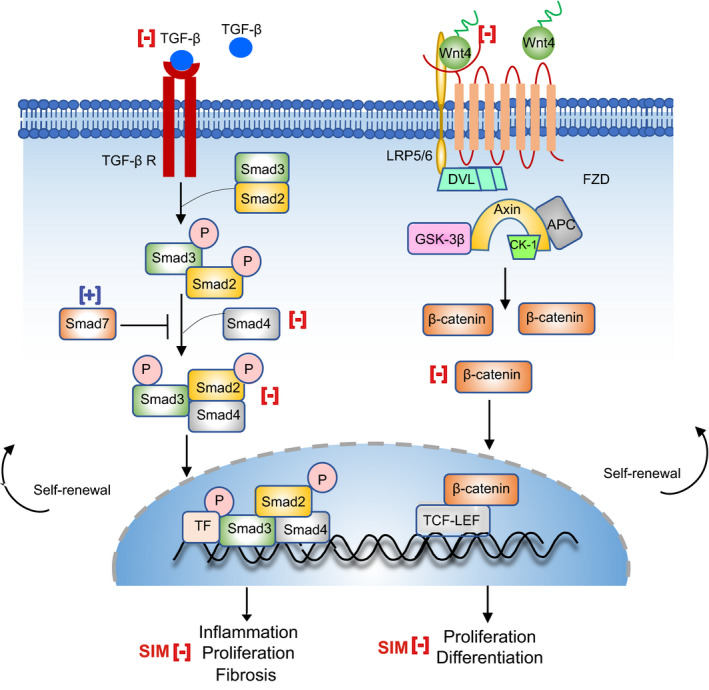
Simvastatin effect on TGF‐β3/SMAD2 and Wnt/β‐Catenin pathways in uterine leiomyoma stem cells. TGF‐β induces their signalling by the TGF‐β receptor. After binding to the receptor, TGF‐β phosphorylates SMAD2 and SMAD3 proteins to form a complex with SMAD4. Phosphorylated SMAD2/3 ties with SMAD4 and moves to the nucleus, regulates the target gene transcription, including SMAD7. SMAD7 is an inhibitory SMAD that blocks SMAD2/3 activation and inhibit NF‐κB‐driven inflammatory response. The absence of Wnt ligands leads to phosphorylation of β‐catenin by the destruction complex that contains the Axin, APC, GSK3‐β and CK1. In this state, β‐catenin is phosphorylated by GSK3β, ubiquitinated and targeted for proteasomal degradation. In the presence of Wnt ligand, Wnt binds to FZD‐LRP5/6 receptor complex, leading to a decrease in β‐catenin degradation in the cytosol, ultimately, increasing the amount of β‐catenin in the nucleus and interacts with LEF/TCF transcription factors, and regulates the expression of an enormous number of genes. Simvastatin's effect on TGF‐β3/SMAD2 and Wnt4/β‐catenin pathways are symbolized by red colour (symbol minus denotes suppression and plus denotes upregulation activity). TGF‐β, transforming growth factor‐β; TF, transcription factor; Wnt, wingless‐type; LRP5/6, lipoprotein receptor‐related protein‐5/6; FZD, frizzled; DVL, dishevelled; APC, adenomatous polyposis coli; CK1, casein kinase 1; GSK3, glycogen synthase kinase 3; LEF, lymphoid enhancer factor; TCF, T‐cell factor

Based on the evidence of simvastatin beneficial effects on UL, as well as the crucial role of stem cells in UL growth, we hypothesized that simvastatin could inhibit tumour growth by targeting tumour stem cells. In this work, we identified an anti‐leiomyoma stem cell effect of simvastatin by inhibiting the TGF‐β3/SMAD2 and Wnt4/β‐catenin signalling pathways involved in proliferation, differentiation and fibrosis **(**Figure 8 **)**.

Compared with normal myometrium tissue, leiomyomas possess fewer stem cells and leiomyoma‐derived stem cell xenografts demonstrate significantly greater proliferation potential than leiomyoma‐derived mature cell xenografts.^4^ In addition to significantly reduced expression of the typical smooth muscle marker α‐SMA and the steroid receptors ER and PR, stem cells contain abundant levels of key stem cell factors Nanog, Sox2 and Oct4. Mature leiomyoma cells exhibit mature phenotypes; there is no significant difference between the mature and total leiomyoma cells in their expression of α‐SMA, ESR1 and PR.

Here, we showed that simvastatin reduces the leiomyoma Stro‐1^+^/CD44^+^ stem cell proliferation compared to control. The suppressed PCNA expression and activated caspase‐3 confirm its antiproliferative and pro‐apoptotic effects on leiomyoma stem cells. In previous works, we demonstrated the anti‐leiomyoma effects of simvastatin using in vitro and in vivo models, including inhibiting proliferation in mature leiomyoma cells by suppressing PCNA expressions,^21^ inducing calcium‐dependent apoptosis,^22^ reducing ECM deposition,^23^ and modulating oestrogen^39^ and mechanotransduction signalling.^26^

Somatic stem cells can be reprogrammed into pluripotent stem cells with self‐renewal, transcriptional function and tumorigenesis potential.^3^, ^4^ In agreement with previous studies, we observed higher Nanog, Oct4 and Sox2 levels, confirming the undifferentiated status of isolated leiomyoma stem cells compared to the mature and total cell populations, and their expression was significantly suppressed by simvastatin.

Fibrosis is one of the hallmarks of leiomyomas, where dysregulated ECM plays a crucial role.^30^, ^31^ ECM such as collagen, fibronectin, laminins and proteoglycan are overexpressed in ULs, which induces mechanotransduction by integrin activation and increases tissue stiffness by altering bidirectional signalling.^31^ Earlier, we observed the inhibition of collagen 1 by simvastatin in leiomyoma cells.^26^ We observed a similar trend of results in the current study in which simvastatin suppressed collagen 1 and fibronectin expression in leiomyoma stem cells.

Several growth factors, inflammatory cytokines and matrix metalloproteinases play a vital role in ECM accumulation and remodelling in ULs.^32^, ^40^ Overproduction of TGF‐β3 is extensively recognized as a key element in tissue fibrosis, involved in the accumulation of collagen 1, fibronectin, laminin and proteoglycan besides induction of leiomyoma cells proliferation and ULs growth.^40^, ^41^ In this study, we found that simvastatin decreases TGF‐β1, 2 and 3 expressions in leiomyoma stem cells. Tissue from UL has been shown to overexpress receptor‐activated SMAD3, common SMAD4 and TGF‐βRs in leiomyoma, compared to the myometrium. Additionally, gonadotropin‐releasing hormone analog (GnRHa) therapy decreases TGF‐βRs, SMAD3 and SMAD4 along with an increase in SMAD7 expression in both myometrium and leiomyoma tissues compared to untreated control.^42^ We demonstrated that simvastatin reduces SMAD2 and SMAD4 expression in leiomyoma stem cells, whereas it increases the expression of the inhibitory SMAD7. In other fibrotic disorders, reduced SMAD7 enhances SMADs signalling and induces inflammation by activating NF‐κB dependent inflammatory response.^36^ In this study, we found that simvastatin significantly decreases the phosphorylation of NF‐κBp65. The increased SMAD7 expression might transiently act as a TGF‐β self‐regulating feedback loop, and a sustainable SMAD7 expression by simvastatin could prolong this inhibitory action. Simvastatin's effect on the TGF‐β3/SMAD2 pathway might explain its inhibitory effect on cell growth and fibrosis in leiomyoma stem cells.

The activation of the Wnt/β‐catenin pathway plays a crucial role in the regeneration and proliferation of UL stem cells to sustain their stem or progenitor properties.^12^ Wnt4, a β‐catenin activator, is highly expressed in *MED12* mutant leiomyoma compared to nonmutant,^11^ and this mutation has been implicated with Wnt/β‐catenin pathway activation and stem cells self‐renewal, proliferation and fibrosis in ULs tissue.^43^ Mature cells release Wnt ligands, which may act on stem cells and induce self‐renewal and proliferation by paracrine fashion.^15^, ^16^ Additionally, Wnt4 enhances the expression of pro‐proliferative genes, c‐Myc and cyclin D1, in leiomyoma stem cells.^37^ Recently, we found that simvastatin reduces the expression of Wnt4 and total β‐catenin, and downstream target of the Wnt/β‐catenin pathway in UL cells.^44^ In the present study, we found that simvastatin treatment significantly reduces the expression of Wnt4 in leiomyoma stem cells. Simvastatin treatment also decreased the mRNA expression of Wnt target genes LRP6, AXIN2, Cyclin D1 and increased APC. β‐catenin plays a vital role in the differentiation of stem cells into the smooth muscle phenotype.^45^ Ono et al. found that nonmutant *MED12* may act as an enhancer of β‐catenin action that supports stem cell renewal, proliferation and fibrosis, whereas mutant or absence may not achieve this action.^12^ Steroid hormone treatment induces nuclear translocation of β‐catenin and their target gene, AXIN2, resulting in the proliferation of leiomyoma stem cells.^12^ In our present study, we found that simvastatin treatment significantly suppresses the β‐catenin expression. We also demonstrated that simvastatin suppresses the expression of a large number of genes and transcription factors in the Wnt pathway.

In summary, the current evidence confirms the presence of tumour stem cells in leiomyoma and support their role in tumour pathogenesis via TGF‐β3/SMAD2 and Wnt/β‐catenin pathways. Herein, we found that simvastatin has a significant inhibitory action on both pathways which may help in suppressing the tumour stem cell niche and, therefore, reduces the continued source of mature tumour cells. Further studies are needed to confirm these findings *in vivo*.

## CONFLICT OF INTEREST

The authors declare no conflict of interest.

## AUTHOR CONTRIBUTIONS

**Sadia Afrin:** Formal analysis (lead); Investigation (lead); Methodology (lead); Software (lead); Validation (lead); Writing – original draft (lead). **Mohamed Ali:** Formal analysis (supporting); Investigation (supporting); Writing – review & editing (supporting). **Malak El Sabeh:** Data curation (supporting); Formal analysis (supporting); Writing – review & editing (supporting). **Qiwei Yang:** Conceptualization (supporting); Formal analysis (supporting); Supervision (supporting); Writing – review & editing (supporting). **Ayman Al‐Hendy:** Conceptualization (supporting); Supervision (supporting); Writing – review & editing (supporting). **Mostafa A. Borahay:** Conceptualization (lead); Data curation (lead); Funding acquisition (lead); Supervision (lead); Writing – review & editing (lead).

## CONSENT TO PARTICIPATE

Written informed consent was obtained from patients.

## CONSENT TO PUBLISH

All authors have given their consent for publication.

## Supporting information

Fig S1

Fig S2

Table S1‐S2

Supplementary Material

## Data Availability

Data and materials will be made available on reasonable request.
