# Supplementary material for: Simvastatin inhibits stem cell proliferation in human leiomyoma via TGF‐β3 and Wnt/β‐Catenin pathways
Source: J Cell Mol Med. 2022 Feb 4;26(5):1684–98. doi: 10.1111/jcmm.17211 (PMC8899165; doi:10.1111/jcmm.17211)
Supplement: Supplementary file 3 — Table S1‐S2 [file JCMM-26-1684-s004.docx]

Supplementary Table 1. Human forward and reverse primers sequence for qRT-PCR.

| Gene | Forward primer sequences | Reverse primer sequences |
| --- | --- | --- |
| *α-SMA* | 5'-GAGCATCCGACACTGCTGAC-3' | 5'-GCACAGCCTGAATAGCCACA-3' |
| *ESR1* | 5′-GCATTCTACAGGCCAAATTCA-3′ | 5′-TCCTTGGCAGATTCCATAGC-3′ |
| *PR* | 5'-GTGGGAGCTGTAAGGTCTTCTTTAA-3' | 5'-AACGATGCAGTCATTTCTTCCA-3' |
| *Nanog* | 5'-CAAAGGCAAACAACCCACTT-'3 | 5'-TCTGCTGGAGGCTGAGGTAT-'3 |
| *OCT4* | 5'-GTATTCAGCCAAACGACCATC-'3 | 5'-CTGGTTCGCTTTCTCTTTCG-'3 |
| *SOX2* | 5'-TACAGCATGATGCAGGACCA-'3 | 5'-CGAGCTGGTCATGGAGTTGTA-'3 |
| *PCNA* | 5'-GCAAGTGGAGAACTTGGAAATG-3' | 5'-GCCTAAGATCCTTCTTCATCCTC-3' |
| *COL1A* | 5'-GGCGACAGAGGCATAAAG-3' | 5'-TCATCAGCCCGGTAGTAG-3' |
| *FN* | 5'-CCATAAAGGGCAACCAAGAG-3' | 5'-AAACCAATTCTTGGAGCA-3' |
| *TGF- β1* | 5'-GGCGATACCTCAGCAACCG -3' | 5'-CTAAGGCGAAAGCCCTCAAT -3' |
| *TGF- β2* | CTGTCTACCTGCAGCACACT | TGGGACTGTCTGGAGCACAA |
| *TGF- β3* | 5'-CACACAGTCCGCTTCTTC-3' | 5'-AGAAGAGGGTGGAAGCC-3' |
| *SMAD2* | 5'-ATGTCGTCCATCTTGCCAATT-3' | 5'-GTCCCCAAATTTCAGAGGAA-3' |
| *SMAD4* | 5'-TGGAATGTAAAGGTGAAGGTGA-3' | 5'-GACACTGACGCAAATCAAAGAC-3' |
| *NF-κB* | 5'-GCCTCCACAAGGCAGCAAATA-3' | 5'-CACCACTGGTCAGAGACTCGGTAA -3' |
| *Wnt4* | 5'-ACCTGGAAGTCATGGACTCG-3' | 5'-TCAGAGCATCCTGACCACTG-3' |
| *β-Catenin* | 5'-AAAATGGCAGTGCGTTAG-3' | 5'-TTTGAAGGCAGTCTGTCGTA-3' |
| *LRP6* | 5'-CATGTGATTGGCTTGGAGAA-3' | 5'-CGACTTGAACCATCCATTCC-3' |
| *AXIN2* | 5'-ACAACAGCATTGTCTCCAAGCAGC-3' | 5'-GCGCCTGGTCAAACATGATGGAAT-3' |
| *Cyclin D1* | 5'-GGATGCTGGAGGTCTGCGAGGAAC-3' | 5'-GAGAGGAAGCGTGTGAGGCGGTAG-3' |
| *APC* | 5'-CAAAACTGGAAACTGAGGCATCT-3' | 5'-ACTCTCCAGAACGGCTTGATACA-3' |
| *PDGFB* | TCCCGAGGAGCTTTATGAGA | ACTGCACGTTGCGGTTGT |
| *PDGFA* | CACACCTCCTCGCTGTAGTATTTA | GTTATCGGTGTAAATGTCATCCAA |
| *IL-1β* | GAAGCTGATGGCCCTAAACA | AAGCCCTTGCTGTAGTGGTG |
| *IL-1α* | TGTATGTGACTGCCCAAGATGAAG | AGAGGAGGTTGGTCTCACTACC |
| *HGF* | GAGAGTTGGGTTCTTACTGCACG | CTCATCTCCTCTTCCGTGGACA |
| *MMP1* | ATGAAGCAGCCCAGATGTGGAG | TGGTCCACATCTGCTCTTGGCA |
| *ITGα3* | GCCTGACAACAAGTGTGAGAGC | GGTGTTCGTCACGTTGATGCTC |
| *TGFBR2* | GTAGCTCTGATGAGTGCAATGAC | CAGATATGGCAACTCCCAGTG |
| *Cav1* | 5′-GTAGACTCGGAGGGACATC-3′ | 5′-CACTTGCTTCTCGCTCAG-3′ |
| *SMAD3* | GTCTGCAAGATCCCACCAG | AGCCCTGGTGACCGACT |
| *STAT6* | CCTTGGAGAACAGCATTCCTGG | GCACTTCTCCTCTGTGACAGAC |
| *SMAD7* | TGTCCAGATGCTGTGCCTTCCT | CTCGTCTTCTCCTCCCAGTATG |
| *CTNNB1* | GCCAAGTGGGTGGTATAGAGG | GGGATGGTGGGTGTAAGAGC |
| *Wnt7B* | AGAAGACCGTCTTCGGGCAAGA | AGTTGCTCAGGTTCCCTTGGCT |
| *Wnt11* | GACCTCAAGACCCGATACCT | GGAGCCCACCTTCTCATTC |
| *GSK3β* | TTCGGGGTCGGAAGACCTTA | GGAACTCCAACAAGGGAGCA |
| *FZD8* | ATCGGCTACAACTACACCTACA | GTACATGCTGCACAGGAAGAA |
| *RhoA* | AGCCTGTGGAAAGACATGCTT | TCAAACACTGTGGGCACATAC |
| *CYP4V2* | AGTTCCAGCCTGAGCGGTTCT | CCTCAGGATGCACGAAAGAATGG |
| *JUN* | CCTTGAAAGCTCAGAACTCGGAG | TGCTGCGTTAGCATGAGTTGGC |
| *DVL1* | GCATAACCGACTCCACCATGTC | GATGGAGCCAATGTAGATGCCG |
| *DVL2* | TCCATACGGACATGGCATCGGT | CGTGATGGTAGAGCCAGTCAAC |
| *Wnt2B* | TGGATGCCAAGGAGAAGAGGCT | GTACAGGAACCACTCACGCCAT |
| *Wnt5A* | CGCCCAGGTTGTAATTGAAG | GCATGTGGTCCTGATACAAGT |
| *FOSL1* | GGAGGAAGGAACTGACCGACTT | CTCTAGGCGCTCCTTCTGCTTC |
| *SOX17* | ACGCTTTCATGGTGTGGGCTAAG | GTCAGCGCCTTCCACGACTTG |
| *TCF7* | GACATCAGCCAGAAGCAAG | CACCAGAACCTAGCATCAAG |
| *Wnt9A* | AGTGCCAGTTCCAGTTCCGCTT | AGGAGATGGCATAGAGGAAGGC |
| *RPLP0* | 5’-GCGACCTGGAAGTCCAACT-3’ | 5’-GGTCCTCCTTGGTGAACAC-3’ |

Supplementary Table 2. List of antibodies with used dilutions.

| Antibodies | Catalog number | Species raised, monoclonal or polyclonal | Technique and dilution used |
| --- | --- | --- | --- |
| α-Smooth muscle actin  (α-SMA) | Cell Signaling technology, #19245 | Rabbit, monoclonal | WB, 1:1000 |
| Estrogen receptor alpha  (ERα) | Invitrogen, # PA5-16440 | Rabbit, polyclonal | WB, 1:500 |
| Progesterone receptor A/B (PR-A/B) | Santa Cruz Biotechnology, sc-166169 | Mouse, monoclonal | WB, 1:500 |
| Nanog | Cell Signaling technology, #4903 | Rabbit, monoclonal | WB, 1:1000; IF, 1:200 |
| Octamer-binding transcription factor 4 (OCT4) | Cell Signaling technology, #75463 | Mouse, monoclonal | WB, 1:1000; IF, 1:200 |
| Sex determining region Y)-box 2 (SOX2) | R&D System, MAB2018 | Mouse, monoclonal | WB, 1:1000; IF, 1:200 |
| Proliferating cell nuclear antigen  (PCNA) | Santa Cruz Biotechnology, sc-7907 | Rabbit, polyclonal | WB, 1:750 |
| Annexin V | Bioss, # bsm-52841R | Rabbit, monoclonal | IF, 1:200 |
| Collagen type I | Santa Cruz Biotechnology, sc-59772 | Mouse, monoclonal | WB, 1:1000 |
| Fibronectin | Abcam, ab6584 | Rabbit, polyclonal | WB, 1:5000 |
| TGF- β1 | Abcam, ab92486 | Rabbit, polyclonal | WB, 1:1000 |
| TGF- β2 | Santa Cruz Biotechnology, sc-374658 | Mouse, monoclonal | WB, 1:500 |
| TGF- β3 | Invitrogen, # PA5-32630 | Rabbit, polyclonal | WB, 1:1000; IF, 1:200 |
| SMAD Family Member 2 (SMAD2) | Cell Signaling technology, #5339 | Rabbit, monoclonal | WB, 1:1000 |
| Phospho-SMAD2 | Invitrogen, # 44-244G | Rabbit, polyclonal | WB, 1:500; IF, 1:200 |
| SMAD4 | Invitrogen, # PA5-34806 | Rabbit, polyclonal | WB, 1:500 |
| SMAD7 | Invitrogen, # 42-0400 | Rabbit, polyclonal | WB, 1:500 |
| Nuclear factor-kappa B p65 (NF-κBp65) | Cell Signaling technology, #8242 | Rabbit, monoclonal | WB, 1:1000 |
| Phospho- NF-κBp65 | Cell Signaling technology, #3033 | Rabbit, monoclonal | WB, 1:1000 |
| Wnt4 | Invitrogen, # 701857 | Rabbit, monoclonal | WB, 1:500; IF, 1:200 |
| β-Catenin | Cell Signaling technology, #8480 | Rabbit, monoclonal | WB, 1:500; IF, 1:200 |
| Beta actin (β-actin) | Sigma-Aldrich, A3854 | Mouse, monoclonal | WB, 1:20000 |

Abbreviations: WB: Western blot, IF, immunofluorescence.
