# Supplementary material for: Simvastatin inhibits stem cell proliferation in human leiomyoma via TGF‐β3 and Wnt/β‐Catenin pathways
Source: J Cell Mol Med. 2022 Feb 4;26(5):1684–98. doi: 10.1111/jcmm.17211 (PMC8899165; doi:10.1111/jcmm.17211)
Supplement: Supplementary file 4 — Supplementary Material [file JCMM-26-1684-s002.docx]

**Supplementary Figure 1 Expression of TGF-β1, 2 and 3 in the leiomyoma mature and stem cells.** The protein expression levels of TGF-β1, 2, and 3 were detected by Western blotting, and β-actin was used as a loading control between two leiomyoma cell populations (mature, M and stem, S). The same Western Blot membrane was probed for TGF-β3 and Wnt4, presented in supplementary figures 1 and 2. Therefore, the same β-actin image was used for all of them. Data are presented as the means ± standard error of the mean (SEM) of the relative expression obtained from three independent experiments.

**Supplementary Figure 2 Expression of Wnt4 in the leiomyoma mature and stem cells.** The protein expression levels of Wnt4 were detected by Western blotting, and β-actin was used as a loading control between two leiomyoma cell populations (mature, M and stem, S). The same Western Blot membrane was probed for TGF-β3 and Wnt4, presented in supplementary figures 1 and 2. Therefore, the same β-actin image was used for all of them. Data are presented as the means ± SEM of the relative expression obtained from three independent experiments.
